# Towards predictive resistance models for agrochemicals by combining chemical and protein similarity via proteochemometric modelling

**DOI:** 10.1007/s12154-014-0112-2

**Published:** 2014-05-15

**Authors:** Gerard J. P. van Westen, Andreas Bender, John P. Overington

**Affiliations:** 1 European Molecular Biology Laboratory European Bioinformatics Institute (EMBL-EBI), Wellcome Trust Genome Campus, Hinxton, Cambridge, CB10 1SD United Kingdom; 2 Unilever Centre for Molecular Informatics, Department of Chemistry, University of Cambridge, Lensfield Road, Cambridge, CB2 1EW United Kingdom

**Keywords:** Polypharmacology, Cheminformatics, Machine learning, Resistance

## Abstract

Resistance to pesticides is an increasing problem in agriculture. Despite practices such as phased use and cycling of ‘orthogonally resistant’ agents, resistance remains a major risk to national and global food security. To combat this problem, there is a need for both new approaches for pesticide design, as well as for novel chemical entities themselves. As summarized in this opinion article, a technique termed ‘proteochemometric modelling’ (PCM), from the field of chemoinformatics, could aid in the quantification and prediction of resistance that acts via point mutations in the target proteins of an agent. The technique combines information from both the chemical and biological domain to generate bioactivity models across large numbers of ligands as well as protein targets. PCM has previously been validated in prospective, experimental work in the medicinal chemistry area, and it draws on the growing amount of bioactivity information available in the public domain. Here, two potential applications of proteochemometric modelling to agrochemical data are described, based on previously published examples from the medicinal chemistry literature.

## Introduction

In agriculture, resistance to pesticides forms a complex and growing problem, which includes the development of resistance to insecticides [17], fungicides [16], as well as herbicides [7, 25, 6]. For each of these resistance types, a multitude of different resistance mechanisms are possible, all of which, however, lead to phenotypic resistance (i.e. the concentration of pesticide needed to kill the pests is higher for resistant variants compared to wild type). Commonly observed resistance mechanisms are similar to those observed in microbial and cancer mechanisms of resistance; examples include increased expression of efflux proteins, increased expression of metabolizing proteins, and point mutations in the protein targeted by the agrochemical agent [8, 10, 24]. Due to the spectrum of possible adaptations in the target organism, it is difficult to capture and model all potential forms of resistance for a certain compound in a model a priori, which is analogous to antibiotic and anti-cancer drug resistance. Out of these possibilities, the current opinion article will deal specifically with the impact of point mutations at the ligand-binding site and their effect on resistance. This is an area for which there is prior successful experience in the medicinal chemistry and drug design field, including prospective experimental validation of the models developed. Here, previous research of the authors as well as other related groups will be outlined, with the aim to transfer these methods also to the world of agrochemical research [22, 20].

## Complementary ligand and target information

**Fig. 1:**
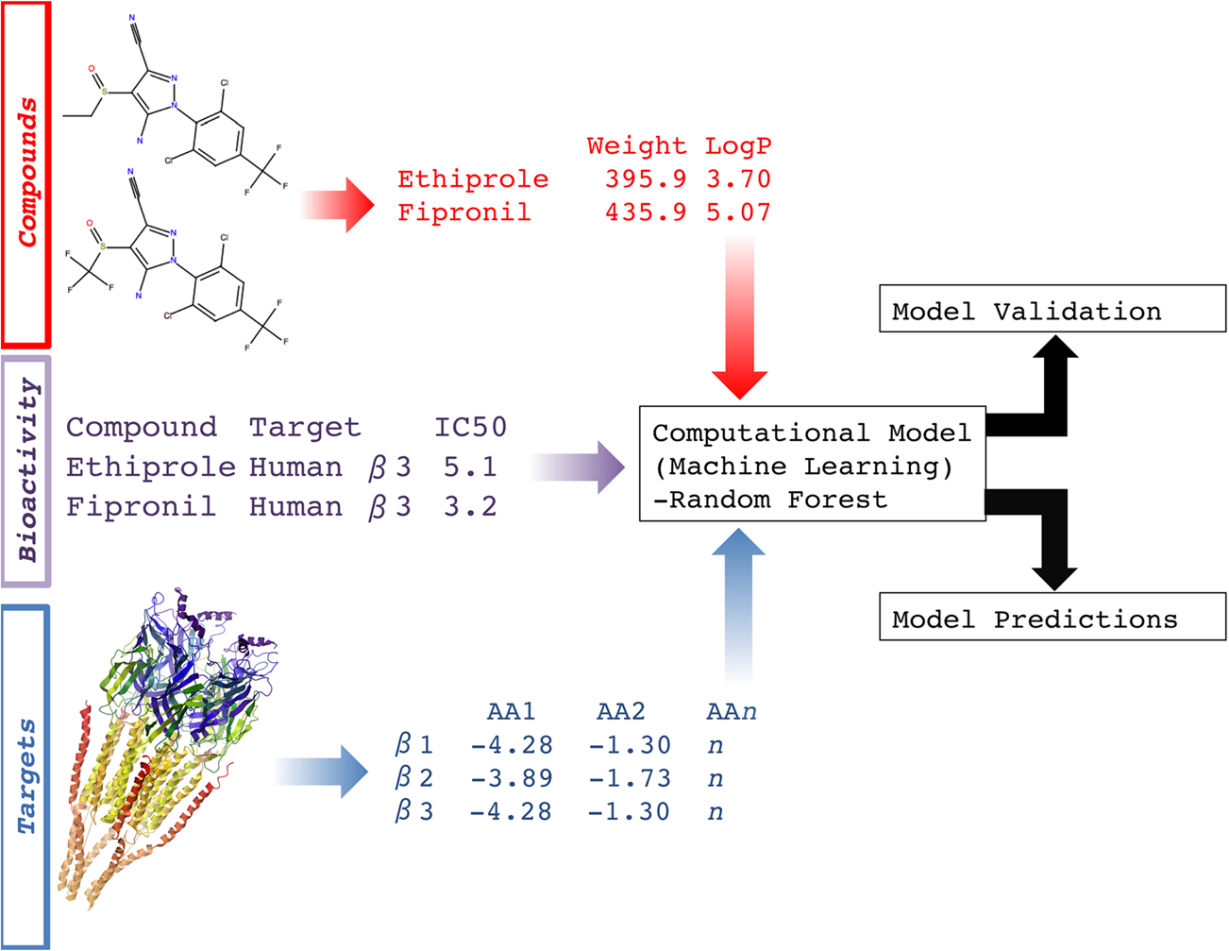
Schematic overview of proteochemometric modelling (PCM). The technique uses chemical properties from multiple ligands (e.g. ethiprole), target properties from multiple proteins (e.g. gamma-aminobutyric acid (GABA) receptor subunit beta-3 [4]), and their respective bioactivity values. From these data, a statistical model is generated. After validation, this model can predict the bioactivity of untested compounds on the targets included in the model, in order to (1) select a compound active on a particular protein target; (2) predict which protein a particular compound will show activity against; or (3) for the prospective selection (‘virtual screening’) of compounds with a desired bioactivity profile against a set of proteins and its mutants

The binding affinity between a ligand (e.g. a small molecule or RNAi) [26, 1], and a target (usually a protein, but potentially a ribosomal target) [18], is governed by properties of both ligand and target (and the physiological environment, e.g. ionic strength and pH). Hence, it follows that changes to either one of the binding partners will, in most cases, translate into a difference (weaker or stronger binding) in affinity and subsequent efficacy. This mechanism is also exploited when a candidate molecule is optimized through iterative design of synthetic molecules to have a high affinity towards a desired protein target via quantitative structure-activity relationship (QSAR) studies, which is a standard technique during lead optimization in drug discovery. In the context of agrochemistry, extending this principle from single targets to multiple targets would be equivalent to optimizing a candidate molecule towards the desired pest, while avoiding unwanted effects, e.g. in humans (as well as other species). This procedure exploits the differences in the sequences and structures between the targets in these species (corresponding to a variation on the target side). In order to now model the activity of multiple ligands against multiple targets, ‘proteochemometric modelling’ (PCM) can be used, a computational technique that simultaneously uses properties of both ligand and target space. Hence, PCM can be used to make predictions for unknown ligands as well as unknown protein targets (Fig. 1; for reviews see [15, 23]). For clarity, these new sequences could be an orthologous sequence from a new pathogen or new sequences generated under selective pressure giving rise to resistant pathogens. The reader is referred to recent reviews and references therein for an overview of the more general concept of computational models of bioactivity and (QSAR) models (these include an overview of various applicable machine learning algorithms and descriptors) [23, 5]. Previously, PCM has been successfully applied to model the impact of protein mutations on the resistance of viruses to drugs. Examples include the human immunodeficiency virus (HIV), also including prospective experimental validation [14, 22, 20], as well as the dengue virus [19]. Given that the problem at hand is similar—in both cases, the impact of sequence mutations/differences of a target protein on ligand binding or efficacy needs to be understood—we anticipate that the concept of PCM will also be transferable to the field of agrochemicals, possibly with some domain-dependent modifications.

## Resistance models

**Fig. 2:**
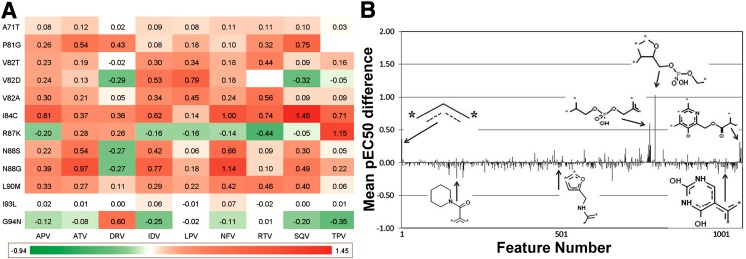
Proteochemometric models can be interpreted biologically (**a**) and chemically (**b**). **a** Displays the average contribution of individual amino acid substitutions to resistance measured as a fold change. Fields are *coloured green* when the combination of mutation and inhibitor is more sensitive, and *red* when the combination is resistant. The *columns* represent clinical HIV protease inhibitors; the *rows* represent point mutations in the following notion: wild-type residue, residue position, and mutated residue (e.g. V82T, valine to threonine at position 82). It can be observed that individual mutations can have different effects on different inhibitors, many of which could be related to experiment (see main text for details). **b** Shows the average contribution of chemical substructures to pEC_50_. The *bars* are positive if the respective feature contributes positively to bioactivity, and negative where the feature leads to lower activity. The *y*-*axis* indicates the average contribution over all present ligands (451) and mutants (14). This information can be used to guide compound selection as well as optimization in cases where bioactivity against multiple protein targets needs to be taken into account. (Fig. adapted from [20] and [22])

The first aim of PCM is to model the bioactivity of ligands against large number of related proteins, which allows the scientist to anticipate the bioactivity spectrum of a molecule in a multidimensional fashion. In the literature, it has been shown that multitarget models combining ligand and target properties (i.e. PCM models) generally perform better than single target models, which are trained on ligand-only properties (i.e. conventional QSAR models) [14, 19, 22, 23, 20]. Moreover, the multitarget nature of these models allows rationalization of resistance, as these models were able to deconvolute the individual contributions of amino acid substitutions (Fig. 2a [20]) or substructures within ligands that vary across a chemical series (Fig. 2b [22]). For instance in the case of HIV protease, it is known that residue 82 can mutate from the wild-type valine to a threonine, and that this simple, conservative, single-point mutation leads to (cross) resistance amongst all clinical inhibitors with the exception of Darunavir [12]. PCM models have been able to reproduce these results, as shown in Fig. 2a.

In the case of agrochemistry, the authors are of the opinion that the nature of the PCM technique would be equally suited to identify potential agrochemicals that have the most favourable resistance profile. Similarly, models could be used to deconvolute contributions of mutations to an increase or reduction of resistance displayed by individual mutants.

## Multispecies models

**Fig. 3:**
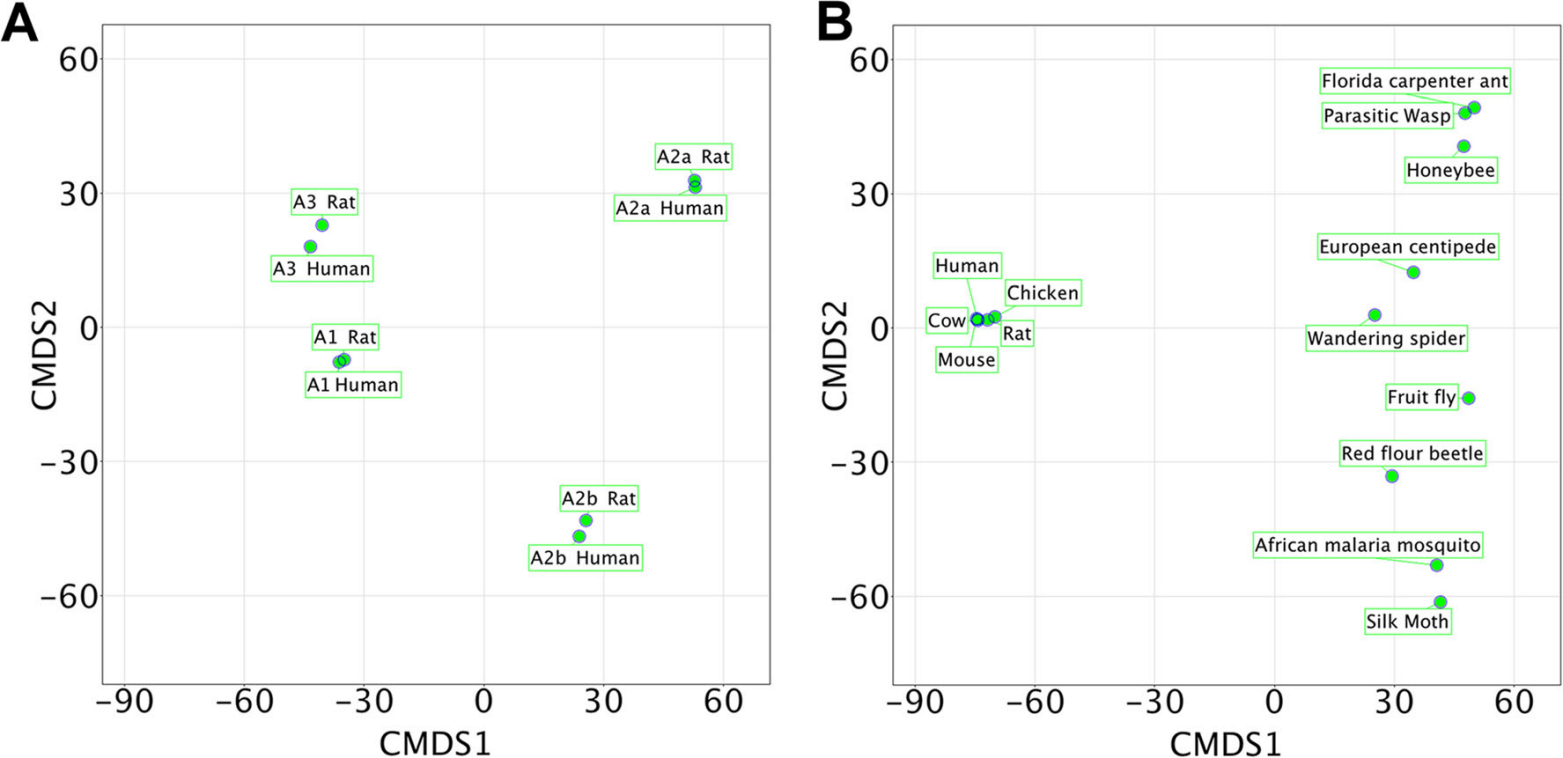
Multidimensional scaling (MDS) plots demonstrating the similarity between protein families in different species. The further points are located from each other, the more dissimilar they are. **a** Full protein similarity between the different adenosine receptor isoforms (orthologues and paralogues) for human and rat. Orthologues (identical function genes in different species) are more similar than paralogues (different function but sequence related genes in the same species). **b** Full similarity between the GABA-A ligand-gated ion channels in mammals and a selection of arthropods. The *plot* illustrates that mammallian receptors are more similar to each other than they are to their insect counterparts, and that insects amongst themselves display a large variation. As it was previously demonstrated that it is possible to model the protein similarity space shown in (**a**) it stands to reason that it is feasible to model the space shown in (**b**). Information of this type can be used (and has been used [21]) in PCM modelling to anticipate, and predict, bioactivity of ligands against orthologues of a protein from multiple species, which is useful for, e.g. off-target modelling

Knowledge on the relationship between homologues targets in different species can be exploited using PCM, which is of relevance either in cases where off-target effects of a compound in a species needs to be avoided, but also importantly where the aim is to target multiple distinct species in a designed spectrum of activity. Previously, a proof-of-concept study [21] employed a single bioactivity model to simultaneously model both the human and rat orthologues of the adenosine receptors (G protein-coupled receptors) using data from the ChEMBL [9] database. The model was able to identify several novel ligands that were experimentally validated, and one of the ligands showed high affinity in the nanomolar range. Upon further inspection, it could be found that the selection of this ligand from a database was likely due to information from other species, underlining the value of integrating as much information from bioactivity space as reasonably possible. In Fig. 3a, a multidimensional scaling analysis (MDS) of the similarity between these eight proteins is shown. From the figure, it is apparent that orthologues (genes closely related in sequence and having the same function in different species) are more similar in the particular definition used than paralogues (genes which are similar in sequence but which have different functions in the same species). Constructing multispecies models on this type of data allows the rationalization of differences in activity between different species, as well as its application for compound design (such as in the above study). As is the case in the application to resistance, this model interpretation can be performed both from the ligand (chemical) point of view and from the target (protein or RNA) point of view, leading on the one hand to the elucidation of chemical features to guide compound optimization, and on the other hand to mutations driving resistance from the protein side.

These approaches are also transferable to the pesticide field. Analogous to the aforementioned adenosine receptors, gamma-aminobutyric acid A (GABA-A) ligand-gated ion channels can be aligned to capture and represent the (dis)similarities between species. These ion channels form the target for phenylpyrazole insecticides, and resistance has been demonstrated through point mutations [2, 4, 3]. An MDS of the similarity between mammalian and insect isoforms of these complexes is shown in Fig. 3b, illustrating that mammalian channels are more similar to each other than they are to their insect counterparts. Furthermore, the selected arthropods display a larger variation between species than do the selected mammals. As it was previously demonstrated that it is possible to model the protein similarity space shown in Fig. 3a (for the adenosine receptors), it stands to reason that it is feasible to model the space shown in Fig. 3b (representing the GABA-A ion channels). PCM models are agnostic of the particular target and application area they are used in—their applicability depends on the amount of data available both from the chemical and biological side (however, this requirement should not be neglected). Hence, the data visualized in Fig. 3b should allow for the construction of a predictive model that can predict activity (and toxicity) of candidate compounds on GABA-A in the species included in the analysis, in a manner similar to the adenosine receptors described above.

Finally, it should be noted that the ribosome has gained significant attention as a druggable target (specifically for antibiotics) [18]. It has been shown that bacteria can gain resistance via multiple diverse mutations in 23S ribosomal RNA [11, 13]. Yet a complementary mode of action leading to novel pesticides should provide a useful addition to established modes of action and increase the resistance threshold.

## Conclusions

In summary, PCM is a versatile quantitative modelling technique for interactions between ligands and their biological targets. In the current opinion paper, we highlight application of the technique and precedence for success from related fields and sketch applications to the agrochemical context, comprising insecticides, fungicides, and herbicides. Based on the prospective experimental validation that has been performed for enzymes and receptors in previous studies, such work is likely to be successful. Firstly, there is the modelling and prediction of resistance towards pesticides by weeds, fungi, or insects. A second application is the prediction of activity of pesticides or other agricultural chemicals in organisms were this is undesired (‘off-targets’). Finally, the technique can be used in virtual screening in the identification of potential new agrochemicals, aimed at a higher resistance threshold (broader activity against multiple mutants), or potential agrochemicals anticipated to have less toxic effects on non-pest species. We anticipate a great future for studies in this area, as the cost of sequencing (which directly relates to the generation of protein-side descriptors) continues to drop and the amount of bioactivity data available increases, both of which increases our ability to generate predictive PCM models considerably.
